# Intrauterine Valvuloplasty in Severe Aortic Stenosis—A Ten Years Single Center Experience

**DOI:** 10.3390/jcm11113058

**Published:** 2022-05-29

**Authors:** Adeline Walter, Brigitte Strizek, Eva Christin Weber, Ingo Gottschalk, Annegret Geipel, Ulrike Herberg, Ulrich Gembruch, Christoph Berg

**Affiliations:** 1 Department of Obstetrics and Prenatal Medicine, University of Bonn, 53127 Bonn, Germany; brigitte.strizek@ukbonn.de (B.S.); annegret.geipel@ukbonn.de (A.G.); ulrich.gembruch@ukbonn.de (U.G.); prof.berg@icloud.com (C.B.); 2 Division of Prenatal Medicine, Gynecological Ultrasound and Fetal Surgery, Department of Obstetrics and Gynecology, University of Cologne, 50937 Cologne, Germany; eva.weber@uk-koeln.de (E.C.W.); ingo.gottschalk@uk-koeln.de (I.G.); 3 Department of Pediatric Cardiology, University of Bonn, 53127 Bonn, Germany; ulrike.herberg@ukbonn.de

**Keywords:** aortic stenosis, congenital heart defect, intrauterine intervention, fetus, prenatal diagnosis

## Abstract

Objective: To assess the course and outcome of fetal aortic valvuloplasty (FAV) in fetuses with severe aortic stenosis (SAS) in a single center. Methods: All fetuses with a prenatal diagnosis of SAS with subsequent FAV were retrospectively collected in one tertiary center for fetal medicine over a period of 10 years. In the study, period fetuses with SAS were considered suitable for FAV in the presence of markedly elevated left ventricular pressures (maximum velocity of mitral regurgitation (MR Vmax) >250 cm/s and/or maximum velocity of aortic stenosis (AS Vmax) >250 cm/s), retrograde flow in the transverse aortic arch and a left ventricular length Z-score >−1. Results: In the study period 29 fetuses with AS were treated with 38 FAV. If reinterventions are included 82.7% of fetuses received a technically successful FAV. Procedure related death occurred in three (10.3%) cases, spontaneous fetal death in 2 (6.9%), and termination of pregnancy was performed in 3 cases (10.3%). Among the 21 live births (72.4%), four died in infancy. Among the remaining survivors, 8/17 (47.1%) had a biventricular outcome at the age of one year, 8/17 (47.1%) were univentricular and one infant (5.9%) is biventricular at the age of eight months. Fetuses with biventricular outcome had significantly greater left ventricular (LV) length Z-scores (*p* = 0.031), and lower tricuspid to mitral valve (TV/MV) ratios (*p* = 0.003). Conclusions: FAV has a high technical success rate and a low rate of procedure related mortality if performed in experienced hands. The success rate of biventricular circulation at the age of one year is moderate and seems to depend rather on the center’s experience and postnatal surgical strategies than solely on prenatal selection criteria. In the absence of randomized controlled trials, FAV remains an experimental intervention.

## 1. Introduction

Severe aortic stenosis (SAS) at midgestation with its typical sonographic features of a normal sized or dilated left ventricle with valvular outflow tract obstruction and markedly elevated left ventricular pressures frequently progresses to hypoplastic left heart syndrome (HLHS) at birth [1,2]. Unfavorable echocardiographic predictors for biventricular circulation are among others left ventricular dysfunction, predominantly retrograde flow in the transverse aortic arch, and left to right flow through the foramen ovale [3].

Fetal aortic valvuloplasty (FAV) was introduced in the 1990s in order to preserve biventricular circulation in fetuses at risk [4]. Since then, albeit still low, the success rate of the interventions improved considerably with 37–43% biventricular (BV) outcome and less than 15% procedure related mortality in high volume centers [5,6].

From initial fetal selection criteria for FAV, as an observation of a progressive disease including also hydrops fetalis in the 1990s, the first accurate inclusion criteria were formed mainly by the working group of Boston and changed during their data acquisition in 2009 [7,8]. Initial inclusion criteria for a technical successful procedure of a biventricular outcome included: left ventricular (LV) long-axis Z-score ≥−2, mitral valve (MV) diameter Z-score >−3, and LV function qualitatively depressed but generating at least a 10 mm Hg pressure gradient across the aortic valve and were changed into LV long-axis Z-score >0, LV short-axis Z-score >0, aortic annulus Z-score >−3.5, MV annulus Z-score >−2 and/or mitral regurgitation (MR) or AS maximum systolic gradient ≥20 mm Hg, achieving a 100% sensitivity for a BV outcome [8]. However, results could not be confirmed by European workgroups, so inclusion criteria were further redefined [2]. Till now recent literature lists selection criteria based mainly on the clinical experience of the two most experienced centers (Boston vs. Linz group). Selection criteria such as a combination of LV pressure >47 mmHg and ascending aorta Z-score ≥0.57 defined by the Boston working group are confronting selection criteria such as a combination RV/LV length ratio <1.09, or alternatively a RV/LV length ratio of 1.09–1.3 combined with MR Vmax >314 cm/s, established by the Linz working group [5,6].

Although the criteria identifying fetuses with SAS that will progress to HLHS are recognized, the criteria [2] identifying those left ventricles that will most likely recover to their full biventricular potential are less well defined. Further, even though the long-term outcome of patients with critical aortic stenosis and biventricular circulation is justifiably assumed to be better than that with univentricular palliation, the risks of biventricular correction are achieved by multiple interventions with increased morbidity and mortality must be considered [5].

In our center, a very simple set of eligibility criteria for FAV in cases with SAS has been applied over the last 10 years. The aim of our current study was to compare the outcomes using these criteria with those from other international centers.

## 2. Materials and Methods

### 2.1. Patients

**Figure 1:**
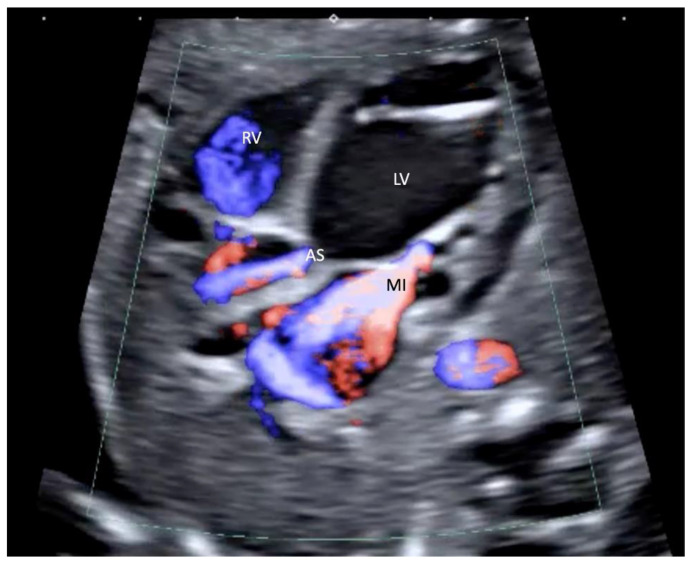
Left outflow tract in a fetus with severe aortic stenosis (AS) at 24 + 2 weeks of gestation. Color Doppler demonstrates a high velocity jet over the aortic valve and massive mitral insufficiency (MI) LV, left ventricle; RV, right ventricle.

**Figure 2:**
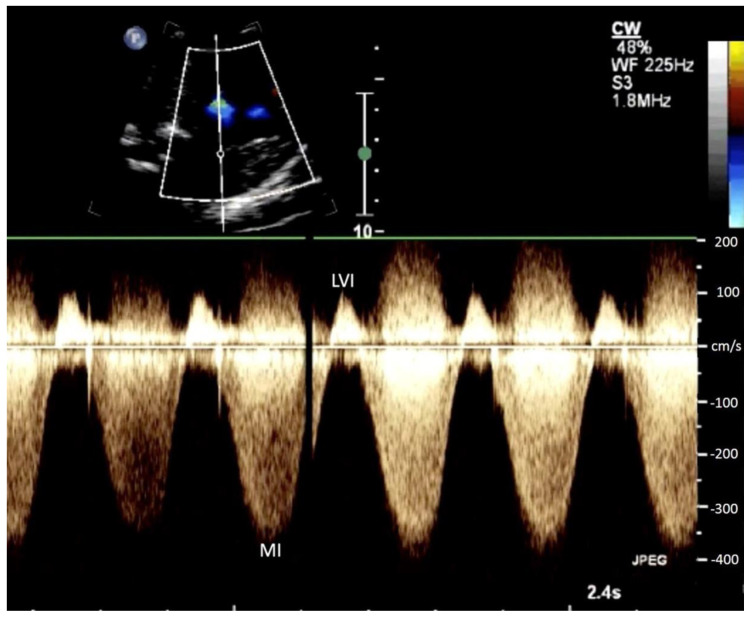
Continuous wave (CW) Doppler of the mitral wave in a fetus at 24 + 4 weeks of gestation with severe aortic stenosis demonstrating still holodiastolic inflow in the left ventricle (LVI) and holosystolic mitral insufficiency (MI) with a maximum velocity of 400 cm/s.

**Figure 3:**
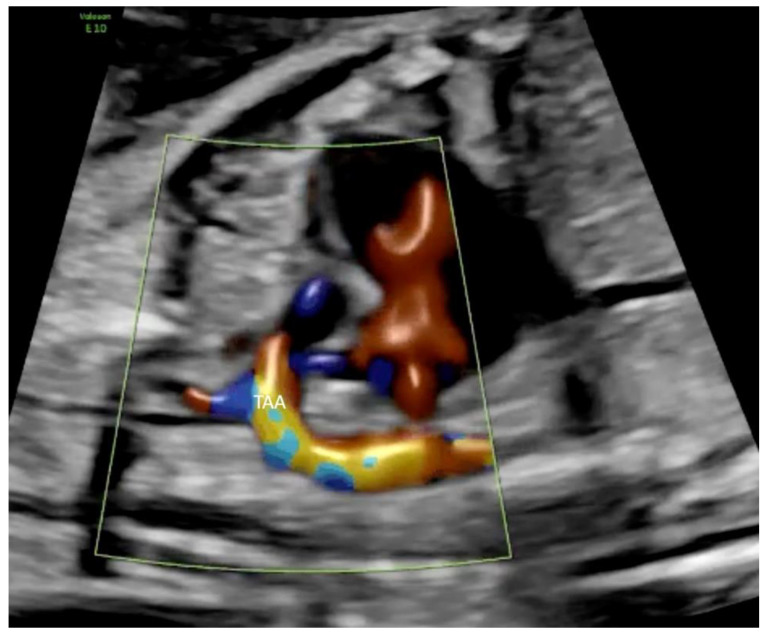
Color Doppler of the transverse aortic arch (TAA) in a fetus at 27 + 3 weeks of gestation with severe aortic stenosis demonstrating holosystolic reversal of flow in the aortic arch.

All cases with a prenatal diagnosis of SAS, defined as a combination of valvular AS (Figure 1), maximum velocity of mitral insufficiency (MI) >250 cm/s, monophasic inflow in the left ventricle (Figure 2), reversal of flow in the transverse aortic arch (Figure 3) and left ventricular length Z-score >−1, detected in a 10 years period (2010 to 2020) in a tertiary referral center (University of Bonn, Bonn, Germany) were retrospectively reviewed for course and outcome. The cut-off value for left ventricular length Z-score >−1, was used, which is in accordance with the recently published data of Tulzer et al. (2022), as using these inclusion criteria seems to be of better prognostic significance for biventricular outcome [5].

For this analysis, we included all fetuses that underwent prenatal intervention. In the study period, all patients received a complete fetal anatomic survey that included fetal echocardiography and Doppler sonography. Ethical approval was granted by the institutional ethics committee of the University of Bonn (Lfd. Nr. 525/20).

### 2.2. Procedure

**Figure 4:**
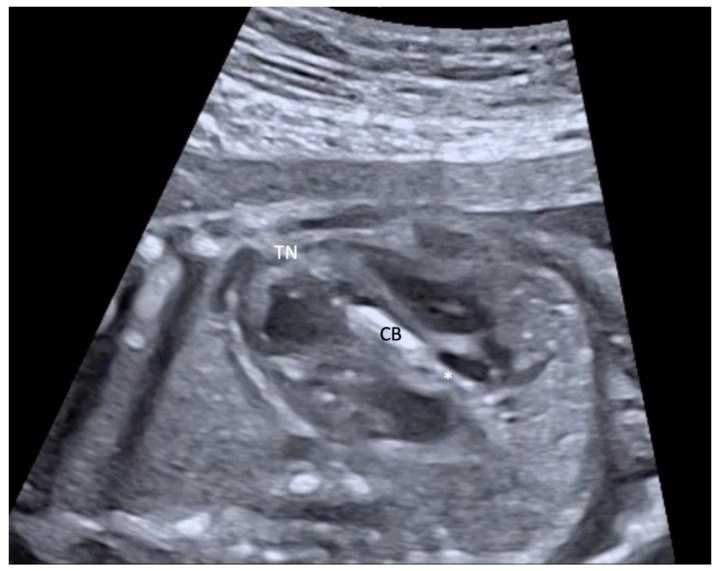
Left outflow tract view in a fetus at 26 + 1 weeks of gestation with severe aortic stenosis with a trocar needle (TN) placed in the left ventricle and the coronary balloon catheter (CB) placed over the aortic valve. The guide wire (asterisk) is positioned in the ascending aorta.

All patients received epidural anesthesia. The fetus was turned to a suitable position by external manipulation, then fetal anesthesia was achieved by intramuscular injection of vecuronium, fentanyl, and atropine in the fetal gluteal muscle as previously described [9]. When fetal immobilization was achieved a trocar needle was advanced under sonographic guidance through the maternal abdomen into the fetal left ventricle. A coronary angioplasty catheter was then advanced over the aortic valve with help of a floppy-tipped guide wire and the balloon was inflated up to rated burst pressure several times (Figure 4).

Due to the long study period, 2 different coronary dilatation catheters 8 mm in length with varying diameters (3.0–4.5 mm), were used: TREK (Abbott Vascular, Santa Clara, CA, USA) and Emerge Monorail (Boston Scientific, Marlborough, MA, USA) applied through an 18 gauge needle (Cook, Bloomington, IN, USA), or 17 gauge needle (Argon Medical Devices, Athens, GA, USA). In the first 3 cases of this series, the balloon size to aortic annulus ratio was 1.0. But in view of the experience of the Linzer working group, published 2010, a balloon size to aortic annulus ratio of 1.2 was chosen in the remaining cases [10].

**Figure 5:**
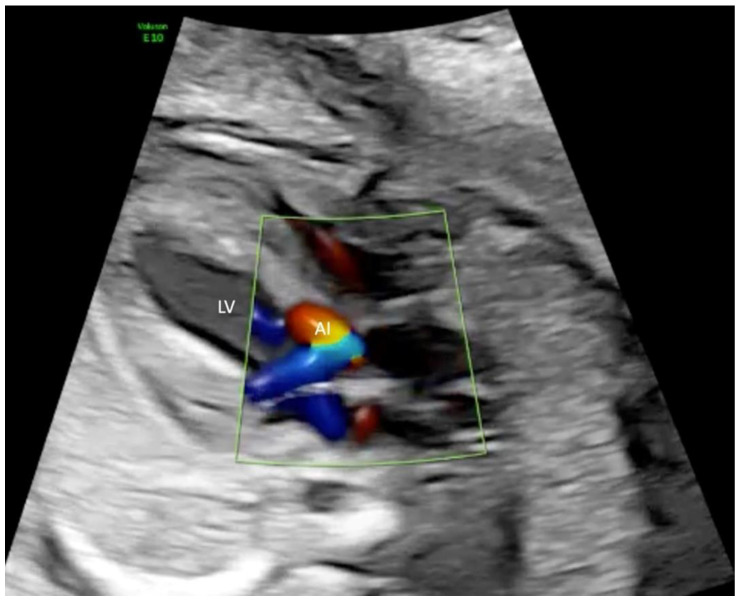
Left outflow tract view in a fetus at 27 + 0 weeks of gestation with severe aortic stenosis after successful balloon dilatation of the aortic valve. Color Doppler demonstrates aortic insufficiency (AI) in diastole. LV, left ventricle.

The procedure was considered technically successful if the balloon was clearly positioned over the aortic valve and if there was improved antegrade flow in the ascending aorta and/or aortic insufficiency (Figure 5).

### 2.3. Outcome

The main outcome measure was the type of postnatal circulation: biventricular circulation was diagnosed if the left ventricle was the sole supplier of systemic output, and if there was no pulmonary hypertension at the age of 1 year. The secondary outcome measure was procedure related loss, defined as intrauterine fetal death at or within 48 h after intervention.

### 2.4. Data Analysis

Measurements of left and right cardiac structures, valves, and areas were performed as described elsewhere. Gestational age-based z-scores were calculated based on data reported by Schneider et al. 2005 and Vignesvaran et al. 2018 [11,12]. Statistical analysis was performed using the Statistical Package for Social Sciences (SPSS 22.0, SPSS Inc., Chicago, IL, USA) statistical software. The independent Student’s test was used for comparisons of continuous data between the two different outcome groups (BV vs. UV). Univariate binary logistic regression analysis was further made to evaluate the prediction of biventricular outcome. Association is expressed by the odds ratio. All values are given as mean ± standard deviation unless indicated otherwise. A *p* value of <0.05 was considered significant.

## 3. Results

**Figure 6:**
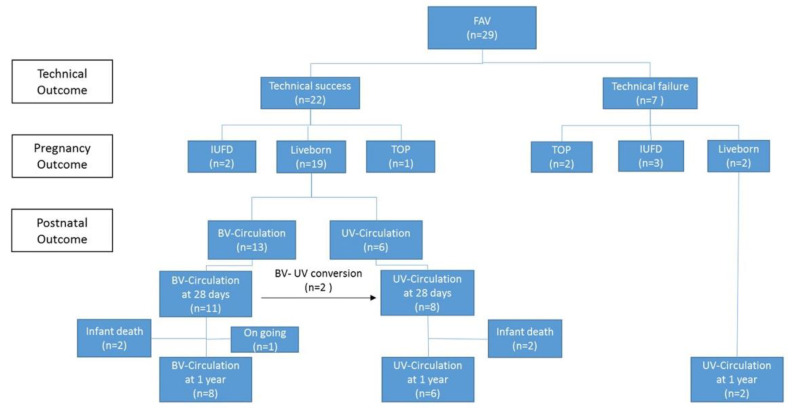
Flowchart summarizing the outcome of 29 fetuses following fetal aortic valvuloplasty.

Between 2010 and 2020, FAV was carried out in 41 fetuses. We excluded 12 cases from the analysis as they had SAS with massive mitral insufficiency, the giant left atrium and intact atrial septum (*n* = 7), hypoplastic left ventricle with premature obstruction of the foramen ovale (*n* = 3), hydrops (*n* = 1) or insufficient image documentation (*n* = 1), leaving 29 cases with isolated SAS for analysis (Figure 6).

The maternal age at intervention was 31.62 (±5.05) with a body mass index (BMI) of 27.38 kg/m^2^ (±6.68). Gestational age at first intervention was 26.14 weeks (±2.56); 23/29 (79.31%) fetuses were male.

In 29 fetuses 38 interventions were performed. In seven fetuses, FAV was repeated 1.41 weeks (±1.19) after the failure of the primary intervention, and in two fetuses, three interventions were performed due to still relevant residual stenosis. The overall technical success rate was 25/38 (65.79%) and improved insignificantly over the study period: 10/16 (62.50%) in 2010–2016 and 16/22 (72.72%) in 2017–2022. If reinterventions are included, 82.7% of fetuses eventually received a technically successful FAV. Factors contributing to the 13 cases of technical failure in 38 procedures included severe bradycardia irresponsive to epinephrine and atropine application and intraventricular erythrocyte transfusion (*n* = 2), leading to a procedure related fetal death in both cases. In another fetus with sustained low cardiac output following FAV, fetal death was registered within 24 h hours after the procedure. Thrombus formation in the LV (*n* = 3), severe pericardial effusion >3 mm requiring repetitive drainage (*n* = 1), or inability to cross the aortic valve (*n* = 5) where further non-lethal factors lead to failure of the intervention. No maternal complications occurred. Procedure related complications are displayed in Table 1.

Intrauterine death occurred in 5/29 (17.24%) cases. Of these, three were directly attributable to a failed intrauterine intervention. In the remaining two cases, initial technical success was achieved, but in one case, closure of the foramen ovale occurred in the further course of pregnancy, and in the other, the stenosis recurred. In both cases, fetal demise was diagnosed weeks after the primary intervention. In 3/29 cases (10.34%) parents opted for termination of pregnancy, two of them due to technical failure and evolving hypoplastic left heart and the third due to closure of the foramen ovale that occurred nearly four weeks after the primary intervention.

21 of 29 (80.77%) fetuses were live born. In two of these, FAV had technically failed and both underwent univentricular palliation and are thriving at the latest follow up. In the remaining 19 newborns with successful FAV initial biventricular (BV) circulation was achieved in 68.42% (13/19). In two of these 13 newborns, BV circulation was converted to UV palliation due to persistent left ventricular dysfunction within the first months of life. Accordingly, univentricular palliation was performed in 8 out of 19 newborns with technically successful FAV.

Six of these eight newborns underwent direct univentricular palliation: 4 of 6 newborns had a Norwood procedure; one of them resulted in unexpected infant death at 157 days after Norwood I and the other 3 completed all stages and are thriving. The remaining two newborns had a hybrid palliation at 68 and 143 days of life, respectively. In one of these two cases, infant death occurred during a comprehensive stage 2 procedure. The other infant is now two years old and in good clinical condition.

Among the 11 children with primary BV circulation after 28 days of life, two died in infancy: one at the age of 29 days after interventional aortic valvuloplasty and later complex surgery with complex commissurotomy of the aortic valve, widening of the left ventricular outflow tract by peeling of endocardial fibroelastosis and reconstruction of mitral valve due to pulmonary edema. In the other case, Shone complex was diagnosed in the neonatal period and infant death occurred at the age of 127 days after mitral valve replacement followed by subsequent graft thrombosis and multiple organ failure.

Among the remaining nine newborns, three neonates had no postnatal intervention up to the last follow up (48 days to 7 years). The remaining six patients underwent initial treatment with aortic balloon valvuloplasty which was followed by surgical interventions in four children: reconstruction of the aortic valve in three children and the Ross-Konno procedure in one child. Therefore, interventional treatment was performed in 66.67% of live born neonates with BV circulation.

If one child that has not yet reached one year of age is excluded, 17/28 (60.1%) fetuses with FAV in our cohort survived to the age of one year; of these 8/17 (47.1%) had a biventricular outcome. Although neither the procedure related mortality nor the BV outcome did improve significantly in the more recent period (2017–2022), there was a marked decrease in neonatal and infant death from 33.33% in 2010–2016 to 8.33% in 2017–2022.

Preinterventional echocardiographic characteristics and univariate analysis of factors predicting biventricular outcome in surviving fetuses with SAS are summarized in Table 2. Fetuses with biventricular outcome had significantly greater LV length Z-scores (*p* = 0.031), and lower TV/MV ratios (*p* = 0.003). Univariate analysis revealed trends toward similar results, although they were only significant for TV/MV Ratio (OR 0.01; 95% CI, 0.1–0.61). Furthermore, univariate analysis revealed significant results for MV Z-scores (*p* = 0.039, OR 1.77, 95% CI, 1.03–3.02), identifying greater scores as predictors associated with BV outcome. Other parameters did not differ significantly, although there was a trend toward greater AoV Dia Z-score (OR 1.81; 95% CI, 0.68–4.80) and greater MR Vmax (cm/s) (OR 1.01; 95% CI, 0.99–1.02) in the biventricular group.

Concerning obstetrical parameters, with the exception of preterm delivery (delivery at 34–37 weeks) (BV: 61.54% vs. UV: 12.50%, *p* = 0.037) and delivery at term (>37 weeks) (BV: 38.46% vs. UV: 87.50%, *p* = 0.035) there were no statistically significant differences between both outcome groups (BV vs. UV).

## 4. Discussion

Since the first reports of successful FAV at the end of the last century, the technical aspects of the intervention have changed very little [4]. In contrast, the criteria of eligibility for FAV have constantly evolved. Ideally, preintervention assessment in SAS has to identify fetuses at high risk for developing HLHS and simultaneously those with the potential of the left ventricle to recover and finally succeed in providing the source of systemic perfusion in a biventricular circulation.

In a retrospective study on the natural history of SAS, a working group from Boston identified a midtrimester combination of moderate to severe left ventricular dysfunction and retrograde flow in the transverse aortic arch and/or left to right flow over the foramen ovale and/or monophasic MV inflow to be present in >90% of fetuses that developed HLHS in the further course of pregnancy [3]. In 2008 the same group published echocardiographic criteria identifying candidates for biventricular outcome after FAV: a combination (4 out of 5) of LV long-axis Z-score (>0), LV short-axis Z-score (>0), aortic annulus diameter Z-score (>−3.5), MV annulus diameter Z-score (>−2), and MR or AS maximum systolic gradient >20 mmHg predicted biventricular outcome with 100% sensitivity [8]. Based on these criteria, however, a European multicenter study on the natural history of SAS showed that 33% of fetuses predicted to evolve to HLHS ended with biventricular circulation, and among those deemed ideal candidates for FAV, 42% had a postnatal biventricular circulation even without fetal intervention [2]. Similar findings were reported by a second European multicenter study including 67 FAV and an adequately matched natural history cohort, that failed to demonstrate improved rates of BV circulation following FAV and thus emphasized the need for better discrimination of candidates [13]. The Boston group recently refined their preprocedural echocardiographic criteria [6]: a combination of LV pressure >47 mmHg and ascending aorta Z-score ≥0.57 had a 92% probability of a biventricular outcome. A working group from Linz very recently published the outcome of 103 FAV and found that the best prediction for the biventricular outcome without signs of PAH at one year of age could be achieved by a combination of cutoffs for the variables RV/LV length ratio and MR Vmax as an LV pressure estimate [5]. The highest rate of biventricular outcome was achieved in cases with a RV/LV length ratio <1.09, or alternatively a RV/LV length ratio of 1.09–1.3 combined with MR Vmax >314 cm/s. The left ventricle pressure estimate was especially important in fetuses prior to 28 weeks gestation as a biventricular outcome could be achieved with even smaller left ventricles if the MR Vmax was high. The authors contribute this to the fact that after successful FAV at an early gestational age there is more time left in utero for the left ventricle to remodel and recover until birth. In our cohort, only the left ventricular dimensions correlated with the outcome, namely LV length Z-score, MV Z-score, and TV/MV Ratio. The LV pressure estimates were not significantly different between biventricular and univentricular outcomes, however, based on our eligibility criteria the whole cohort already had high LV pressures at inclusion, which impairs the predictive value of this parameter.

The procedure related fatality of FAV varies largely between centers and is most likely associated with the caseload, with lower loss rates in high volume centers. A recent meta-analysis of seven studies including 266 fetuses with FAV reported a fetal loss rate of 16% (range 0−32%) [14]. The largest published series from Boston [6] and Linz [5] reported procedure related loss rates of 8% and 11%, respectively. These latter publications cover study periods of over 15 years and report significantly improved survival rates over time. Although our cohort is significantly smaller, the procedure related loss rate of 10% is comparably low, however, due to the small number of interventions these data are not very robust. Together with the limited number of cases, this is probably the explanation why we failed to demonstrate a learning curve over the study period.

The two largest cohorts published to date from Boston [6] and Linz [5] reported overall 36.6% and 42.7% biventricular circulation after one year, respectively. If only live born children with technically successful FAV were considered, the figures in these cohorts improved to 45.2% and 55.0%, respectively. The BV-rate in our cohort is somewhat lower with 28.6% overall and 47.1% if only technically successful survivors are considered. The biggest difference between these two studies and ours is the caseload. Both centers are performing FAV for over 20 years and have each performed over 100 interventions. A Spanish working group with nine-year experience and 28 cases which is very similar to our cohort reported 21.4% overall biventricular outcome and 54.5% if only live born children with successful interventions were considered [15]. In this latter cohort however, the procedure related loss rate and the number of terminations were over 50%, therefore the numbers have to be carefully considered. In the meta-analysis by Vorisek et al. [14], the overall biventricular outcome was 36.8% and 52% if only live born children with successful interventions were considered.

Our selection criteria are unlikely to be the reason for the lower success rate, as they didn’t change over the study period and largely correspond to the recently published Linz criteria [5]. However, the outcome of the postnatal circulation is also influenced by different surgical strategies and clinical decision-making. This is supported by the fact that in the recently published series up to 52% of cases received an early Ross-Konno procedure [5], in contrast to only 11% in our cohort. According to the strategy of our center, we perform a Ross-Konno procedure at the earliest at an age of three months and proceed with a repeat dilation of the aortic valve for residual stenosis and a commissurotomy and reconstruction of the aortic valve in case of relevant aortic regurgitation. However, the implementation of an early Ross-Konno procedure may have a clinical impact with regards to the fetal and neonatal outcome. It may enable biventricular repair even in borderline left ventricles with a good long-term outcome and should be further discussed with regard to FAV [16]. In addition, initial hybrid palliation can stimulate left heart growth in some patients with left ventricle hypoplasia and may eventually achieve biventricular circulation in some of them [17].

In addition, endocardial fibroelastosis and mitral valve involvement have an important impact on postnatal surgical strategies and outcomes.

## 5. Conclusions

FAV has a high technical success rate and a low rate of procedure related mortality if performed in experienced hands. The success rate of biventricular circulation at the age of one year is moderate and seems to depend rather on the center’s experience and postnatal surgical strategies than on prenatal selection criteria. In the absence of randomized controlled trials, FAV remains an experimental intervention.

## Figures and Tables

**Table 1:** Complications of FAV procedures (*n* = 38), categorized by technical success.

Complication	Successful (25/38)	Not Successful (13/38)	Over All (%)
Bradycardia	2	2	10.53
Pericardial effusion	3	1	10.53
Drainage of pericardial effusion	1	1	5.26
Epinephrine	1	2	7.89
Thrombus in left ventricle	0	3	7.89
Intrauterine blood transfusion	2	2	10.53
Premature Labor (without preterm delivery)	2	0	5.26
Procedure related fetal death	0	3	7.89

**Table 2:** Preinterventional echocardiographic characteristics and univariate analysis of factors for biventricular circulation outcome of live born fetuses with CAS prior to fetal aortic valvuloplasty (FAV).

Parameter	All Liveborn	Biventricular (*n* = 13)	Univentricular (*n* = 8)	Odds Ratio (95% CI)	*p*–Value (OR)	*p*-Value (Student)
LV Z-score	−0.31 (±1.05)	0.1 (±0.86)	−0.8 (±1.06)	4.0 (0.92–17.21)	0.063	0.031
RV/LV length Ratio	0.93 (±0.13)	0.88 (±0.93)	0.97 (±0.16)			0.053
MV Z-score	−2.58 (±3.35)	−1.9 (±3.58)	−3.58 (±2.9)	1.77 (1.03–3.02)	0.039	0.287
TV/MV Ratio	1.37(±0.49)	1.14 (±0.12)	1.77 (±0.62)	0.01 (0.1–0.61)	0.032	0.003
AoV ^1^ Diameter Z-score	3.44 (±1.09)	−3.21 (±1.17)	−3.84 (±0.88)	1.81(0.68–4.80)	0.237	0.243
Aortic stenosis (mmHg)	34.45 (±15.83)	39.77 (±14.58)	18.50 (±3.54)			0.100
MR Vmax (cm/s)	379.61 (±76.11)	390.91 (±58.55)	359.29 (±99.94)	1.01 (0.99–1.02)	0.385	0.407
Pulmonary venous flow pulsatility		5 (normal) 5 (moderate) 2 (severe)	3 (normal) 1 (moderate) 3 (severe)			
GA at intervention (weeks)	26.59 (±2.51)	27.31 (±2.77)	25.63 (±1.85)	1.41 (0.88–2.25)	0.154	0.132

^1^ AoV, aortic valve; GA, gestational age; LV, left ventricle; MR, mitral regurgitation; MV, mitral valve; RV/LV, right ventricle/left ventricle; TV/MV, tricuspid valve/mitral valve.

## Data Availability

The data presented in this study are available on request from the corresponding author.

## References

[1] Simpson J.M., Sharland G.K. (1997). Natural history and outcome of aortic stenosis diagnosed prenatally. Heart.

[2] Gardiner H.M., Kovacevic A., Tulzer G., Sarkola T., Herberg U., Dangel J., Öhman A., Bartrons J., Carvalho J.S., Jicinska H. (2016). Fetal Working Group of the AEPC. Natural history of 107 cases of fetal aortic stenosis from a European multicenter retrospective study. Ultrasound Obstet. Gynecol..

[3] Mäkikallio K., McElhinney D.B., Levine J.C., Marx G.R., Colan S.D., Marshall A.C., Lock J.E., Marcus E.N., Tworetzky W. (2006). Fetal aortic valve stenosis and the evolution of hypoplastic left heart syndrome: Patient selection for fetal intervention. Circulation.

[4] Maxwell D., Allan L., Tynan M.J. (1991). Balloon dilatation of the aortic valve in the fetus: A report of two cases. Heart.

[5] Tulzer A., Arzt W., Gitter R., Sames-Dolzer E., Kreuzer M., Mair R., Tulzer G. (2022). Valvuloplasty in 103 fetuses with critical aortic stenosis: Outcome and new predictors for postnatal circulation. Ultrasound Obstet. Gynecol..

[6] Friedman K.G., Sleeper L.A., Freud L.R., Marshall A.C., Godfrey M.E., Drogosz M., Lafranchi T., Benson C.B., Wilkins-Haug L.E., Tworetzky W. (2018). Improved technical success, postnatal outcome and refined predictors of outcome for fetal aortic valvuloplasty: Biventricular outcome after fetal aortic valvuloplasty. Ultrasound Obstet. Gynecol..

[7] Kohl T., Sharland G., Allan L.D., Gembruch U., Chaoui R., Lopes L.M., Zielinsky P., Huhta J., Silverman N.H. (2000). World experience of percutaneous ultrasound-guided balloon valvuloplasty in human fetuses with severe aortic valve obstruction. Am. J. Cardiol..

[8] McElhinney D.B., Marshall A.C., Wilkins-Haug L.E., Brown D.W., Benson C.B., Silva V., Marx G.R., Mizrahi-Arnaud A., Lock J.E., Tworetzky W. (2009). Predictors of technical success and postnatal biventricular outcome after in utero aortic valvuloplasty for aortic stenosis with evolving hypoplastic left heart syndrome. Circulation.

[9] Tworetzky W., Wilkins-Haug L., Jennings R.W., van der Velde M.E., Marshall A.C., Marx G.R., Colan S.D., Benson C.B., Lock J.E., Perry S.B. (2004). Balloon dilation of severe aortic stenosis in the fetus: Potential for prevention of hypoplastic left heart syndrome: Candidate selection, technique, and results of successful intervention. Circulation.

[10] Arzt W., Wertaschnigg D., Veit I., Klement F., Gitter R., Tulzer G. (2011). Intrauterine aortic valvuloplasty in fetuses with critical aortic stenosis: Experience and results of 24 procedures. Ultrasound Obstet. Gynecol..

[11] Schneider C., McCrindle B.W., Carvalho J.S., Hornberger L.K., McCarthy K.P., Daubeney P.E.F. (2005). Development of Z-scores for fetal cardiac dimensions from echocardiography. Ultrasound Obstet. Gynecol..

[12] Vigneswaran T.V., Akolekar R., Syngelaki A., Charakida M., Allan L.D., Nicolaides K.H., Zidere V., Simpson J.M. (2018). Reference ranges for the size of the fetal cardiac outflow tracts from 13 to 36 weeks gestation: A single-center study of over 7000 cases. Circ. Cardiovasc. Imaging.

[13] Kovacevic A., Öhman A., Tulzer G., Herberg U., Dangel J., Carvalho J.S., Fesslova V., Jicinska H., Sarkola T., Pedroza C. (2018). the Fetal Working Group of the AEPC. Fetal hemodynamic response to aortic valvuloplasty and postnatal outcome: A European multicenter study: Fetal hemodynamic response to aortic valvuloplasty. Ultrasound Obstet. Gynecol..

[14] Vorisek C.N., Zurakowski D., Tamayo A., Axt-Fliedner R., Siepmann T., Friehs I. (2022). Postnatal outcome in patients with aortic stenosis undergoing fetal aortic valvuloplasty: Systematic review and meta-analysis. Ultrasound Obstet. Gynecol..

[15] Galindo A., Comas C., Martínez J.M., Gutiérrez-Larraya F., Carrera J.M., Puerto B., Borrell A., Mortera C., de la Fuente P. (2003). Cardiac defects in chromosomally normal fetuses with increased nuchal translucency at 10–14 weeks of gestation. J. Matern.-Fetal Neonatal Med..

[16] Sames-Dolzer E., Wickenhauser E., Kreuzer M., Benedikt P., Gitter R., Prandstetter C., Gierlinger G., Tulzer G., Mair R. (2018). The Ross-Konno procedure in neonates and infants less than 3 months of age. Eur. J. Cardiothorac. Surg..

[17] Sojak V., Bokenkamp R., Kuipers I., Schneider A., Hazekamp M. (2021). Left heart growth and biventricular repair after hybrid palliation. Interact Cardiovasc. Thorac. Surg..

